# Scutellarin Reduce the Homocysteine Level and Alleviate Liver Injury in Type 2 Diabetes Model

**DOI:** 10.3389/fphar.2020.538407

**Published:** 2020-12-11

**Authors:** Yiyu Wang, Xiaoming Fan, Biao Fan, Kerong Jiang, Haoxin Zhang, Feng Kang, Hui Su, Danshan Gu, Shude Li, Shaofang Lin

**Affiliations:** ^1^ Department of Biochemistry and Molecular Biology, School of Basic Medicine, Kunming Medical University, Kunming, China; ^2^ Department of Clinical Laboratory, Affiliated Sichuan Provincial Rehabilitation Hospital of Chengdu University of TCM, Sichuan, China; ^3^ Guangxi Key Laboratory of Diabetic Systems Medicine, Guilin Medical University, Guilin, China; ^4^ The Center of Basic Experiment, School of Basic Medicine, Kunming Medical University, Kunming, China; ^5^ Department of Pharmacology, School of Basic Medicine, Kunming Medical University, Kunming, China; ^6^ Yunnan Province Key Laboratory for Nutrition and Food Safety in Universities, Kunming, China; ^7^ Department of Geriatrics, Gan Mei Hospital, The First People Hospital of Kunming City, Kunming, China

**Keywords:** scutellarin, type 2 diabetes, Hcy, liver tissue, apoptosis

## Abstract

Scutellarin (SCU) is an active ingredient extracted from *Erigeron breviscapus (Vaniot) Hand.-Mazz*. Its main physiological functions are anti-inflammatory and antioxidant. In this study, we established a STZ-induced model of type 2 diabetes (T2DM) and a homocysteine (Hcy)-induced apoptosis model of LO2 to investigate whether SCU can alleviate liver damage by regulating Hcy in type 2 diabetes. Biochemical analysis indicated that SCU could improve the lipid metabolism disorder and liver function in diabetic rats by downregulating the levels of triglycerides (TG), cholesterol (CHO), low-density lipoprotein (LDL), alanine transaminase (ALT) and aspartate transaminase (AST), and by upregulating the level of high-density lipoprotein (HDL). Interestingly, SCU also could down-regulate the levels of Hcy and insulin and enhance the ability of type 2 diabetic rats to regulate blood glucose. Mechanistically, our results indicated that SCU may control the level of Hcy through regulating the levels of β-Cystathionase (CBS), γ-Cystathionase (CSE) and 5,10-methylenetetrahydrofolate (MTHFR) in liver tissue, and up-regulate folic acid, VitB_6_ and VitB_12_ levels in serum. Furthermore, SCU inhibits apoptosis in the liver of T2DM rats and in cultured LO2 cells treated with Hcy. Together, our findings suggest that SCU may alleviate the liver injury thorough downregulating the level of Hcy in T2DM rats.

## Introduction

As the changes of living standard and the dietary structure, there are approximately 425 million people worldwide with diabetes (Hallberg et al., 2019b). The diseases induced by these factors have now spread all over the world, and have no trend of remission (Hallberg et al., 2019a). Currently, approximately 79% of adults with diabetes live in low- and middle-income countries (Misra et al., 2019). Among these complications, vascular lesions are an important factor in a series of damage to tissues and organs (Wen et al., 2019). Among them, the probability of death from diabetes patients due to cardiovascular complications is 3–4 times that of non-diabetics (Monnier et al., 2019). Numerous studies have shown that homocysteine (Hcy) is involved in the development and progression of many vascular complications (Hasan et al., 2019).

Hcy is an intermediate of methionine metabolism and is metabolized in the body mainly through the remethylation pathway and the sulfur transfer process. The concentration of Hcy is closely related to the exon polymorphism C677T of methylentetrahydrofolate reductase (MTHFR) gene, and the level of Hcy in human serum carrying 677T allele tends to increase (Huang et al., 2018). In patients with type 2 diabetes, methionine-methylation is weakened, and decreased clearance of Hcy is one of the causes of hyperhomocysteinemia (HHcy) in patients with type 2 diabetes (Wijekoon et al., 2005). For every 5 μmol/L increase in circulating Hcy concentration, the risk of diabetic nephropathy increases by about 4 times (Ma et al., 2019). Domestic and foreign scholars hold the same view on the relationship between Hcy and diabetes, and believe that the serum Hcy level of diabetic patients is higher than that of normal people. Hcy increases the risk of oxidative stress damage (Dos Santos et al., 2019). Hcy can also cause apoptosis through two pathways, endoplasmic reticulum stress and mitochondrial stress (Zhang et al., 2017b).

Scutellarin (SCU) is an active ingredient extracted from the *Erigeron breviscapus (Vaniot) Hand.-Mazz*. It has been made into capsules and injections. Most studies have demonstrated that anti-inflammatory and antioxidant are the main biological activities of SCU (Mo et al., 2018). It has been reported that SCU can improve diabetes mellitus inflammation through Nrf2/HO-1 signaling pathway (Liu et al., 2019). Furthermore, Zhang et al. (Zhang et al., 2017a) found that SCU not only inhibits the inflammatory response, but also down-regulates the levels of caspase-3 and caspase-9 in arthritic rats. It indicated that SCU can alleviate the occurrence of apoptosis to some extent.

In this study, we established a model of type 2 diabetes and Hcy-induced apoptosis in LO2 cells to study the effect of SCU on the content of Hcy and the changes of its metabolic pathway-related factors in type 2 diabetes, and to explore whether SCU can alleviate the apoptosis of liver cells in type 2 diabetes. This study will provide a scientific basis for SCU treatment of type 2 diabetes and its complications.

## Methods

### Reagents and Antibodies

SCU (purity 98%) was purchased from Honghe Qianshan Bioengineering Co., Ltd. (Honghe, China). Streptozocin (STZ) (T1507) and Rosiglitazone (2A-11884-1) were obtained from Multi Sciences (lianke) Biotech, CO. LTD. (Hangzhou, China). Antibodies to detect β-actin (#4970), Cleaved caspase-3 (#9664), procaspase-3 (#12742), cleaved caspase-3 (#9579), CES (#19689), CBS (#22273), MTHFR (#25164), MTR (# 68796S) were ordered from Cell Signaling Technology (Danvers, MA, United States). The ELISA kit to detect Hcy (#E031-1-1), VitB6, VitB12 and Folic acid were purchased from Nanjing Jiancheng Bioengineering Institute (Nanjing, China).

### Construction and Grouping of Animal Models

Adult male SD rats (180–200 g), obtained from the Experimental animal center of Kunming Medical University, China, were kept under standardized conditions: room temperature, 22 ± 2°C; relative humidity 45–55%; 12-h light/dark cycle in the animal facility with free access to food and water.

Two weeks after the adaptive feeding of the rats, the normal group continued to give a normal diet. The remaining rats were fed with high-fat, high-sugar (HFHS) diet (15% lard, 30% sucrose, 2% cholesterol, 1% sodium cholate, 5% protein powder, and 47% regular diet). After 8 weeks of feeding, rats were injected with 35 mg/kg STZ intraperitoneally to induce the model of type II diabetes, and then treated with normal diet until the end of the experiment. STZ was freshly prepared in ice-cold citrate buffer (0.1 M, pH 4.35). Animals whose fasting blood sugar level was higher than 16.7 mmol/L three days after STZ injection were successfully moulded.

After seven days of the injection of STZ, oral administration of SCU and Rosiglitazone was treated the rats at the dose of 100 (Low dose, LD) or 200 mg/kg/d High dose, HD) and 5 mg/kg/d Rosiglitazone during 8 weeks.

### Cell Culture

Human normal LO2 liver cells were ordered from American Type Culture Collection. The cells were cultured at 37°C in a 5% CO_2_ incubator with RPMI1640 and 10% fetal bovine serum. LO2 cells were pretreated with SCU for 4 h and then induced by 1.5 mM Hcy for 24 h.

### Biochemical Index Detection

FBG levels were measured by Glucose meter (Accu-Check Performa, Germany). TG and CHO levels were measured by automatic biochemical analyzer. The levels of insulin, homocysteine, folic acid, vitamin B_6_, vitamin B_12_, IL-1, IL-6, TNF-a were detected by ELISA kit. The level of MDA, SOD and GSH was determined by Thiobarbituric acid (TBA) method, hydroxylamine method and microplate method respectively by following the instructions of manufacturer.

### H&E, Immunohistochemical and Terminal deoxynucleotidyl transferase dUTP nick end labeling Staining

Taken a small piece of tissue and fixed it in 10% paraformaldehyde. After washing with tap water, it was subjected to a series of operations such as dehydration, transparent, paraffin embedding and sectioning for HE staining. For Immunohistochemical detection, paraffin sections were repaired with citric acid, antibody was added to each section respectively (CBS 1:50; CSE 1:500; MTHFR 1:100; MTR 1:100; Caspase-3 1:1,000) at 4°C incubation overnight. Secondary antibody was added and incubated at room temperature for 15 min. After staining with hematoxylin for 10 min, it was differentiated with ethanolic hydrochloric acid for 20 s and rinsed with water for 10 min. After dehydration with ethanol, it was made transparent with xylene, sealed with a neutral gel. For TUNEL staining, after dewaxing the liver paraffin section, the section was added to the proteinase K for 30 min, and the mixture solution of TdT enzyme and Biontin-dUTP (TdT: Biontin-dUTP = 1:9) was incubated in 37°C for 1 h, then the Converter-AP solution was added in 37°C incubate for 20 min; The BCIP/NBT solution and the nucleus red were subjected to a color reaction, and the AEC aqueous sealing tablets were used for sealing.

### Western Blotting

Total protein was extracted with RIPA lysate added with PMSF, then quantified by BCA and finally the protein concentration was pulled to 5 μg/μl. The protein was loaded at 40 μg, and the protein was separated by SDS-PAGE 8% gel and transferred to a polyvinylidene fluoride (PVDF) membrane and blocked with 5% milk for 120 min at room temperature. Then, the membrane was incubated with CBS polyclonal antibody (1:1,000), CSE polyclonal antibody (1:4,000), MTR polyclonal antibody, MTHFR polyclonal antibody (1:2,000), Cleaved caspase-3 polyclonal antibody (1:1,000), procaspase-3 polyclonal antibody (1:1,000) and β-actin polyclonal antibody (1:4,000) overnight at 4°C.

### CCK-8 Assay

LO2 cells (1 * 10^3^ cells per well) were seeded in a 96-well plate, and treated with 0.5, 1.0, 1.5, 2.0, and 3.0 mM Hcy, or 0.1, 0.3, 0.9 mM SCU respectively. The cytotoxicity of SCU was measured at 24 h using a CCK8 assay kit. The absorbance was read at 450 nm according to the instructions.

### Apoptosis Assay

LO2 cells were pretreated with 0.1, 0.3, 0.9 mM SCU for 4 h, and then induced with 1.5 mM Hcy for 24 h. After treatment, the cell culture solution and adherent cells were collected into a 15 ml centrifuge tube, centrifuged at 1,000 rpm for 5 min, the supernatant was poured out, and 500 μl buffer was added to the tube in order to suspend the cells. The data was measured by flow cytometer according to the instruction.

### Statistical Analysis

Statistical analysis was conducted using SPSS 13.0 software. The data were measured by mean ± standard deviation (x ± s). One-way ANOYA was used for comparison among groups. *p* < 0.05 indicating that the difference was statistically significant.

## Results

### Scutellarin Improves Dyslipidemia and Liver Function in Type 2 Diabetic Rats

**FIGURE 1:**
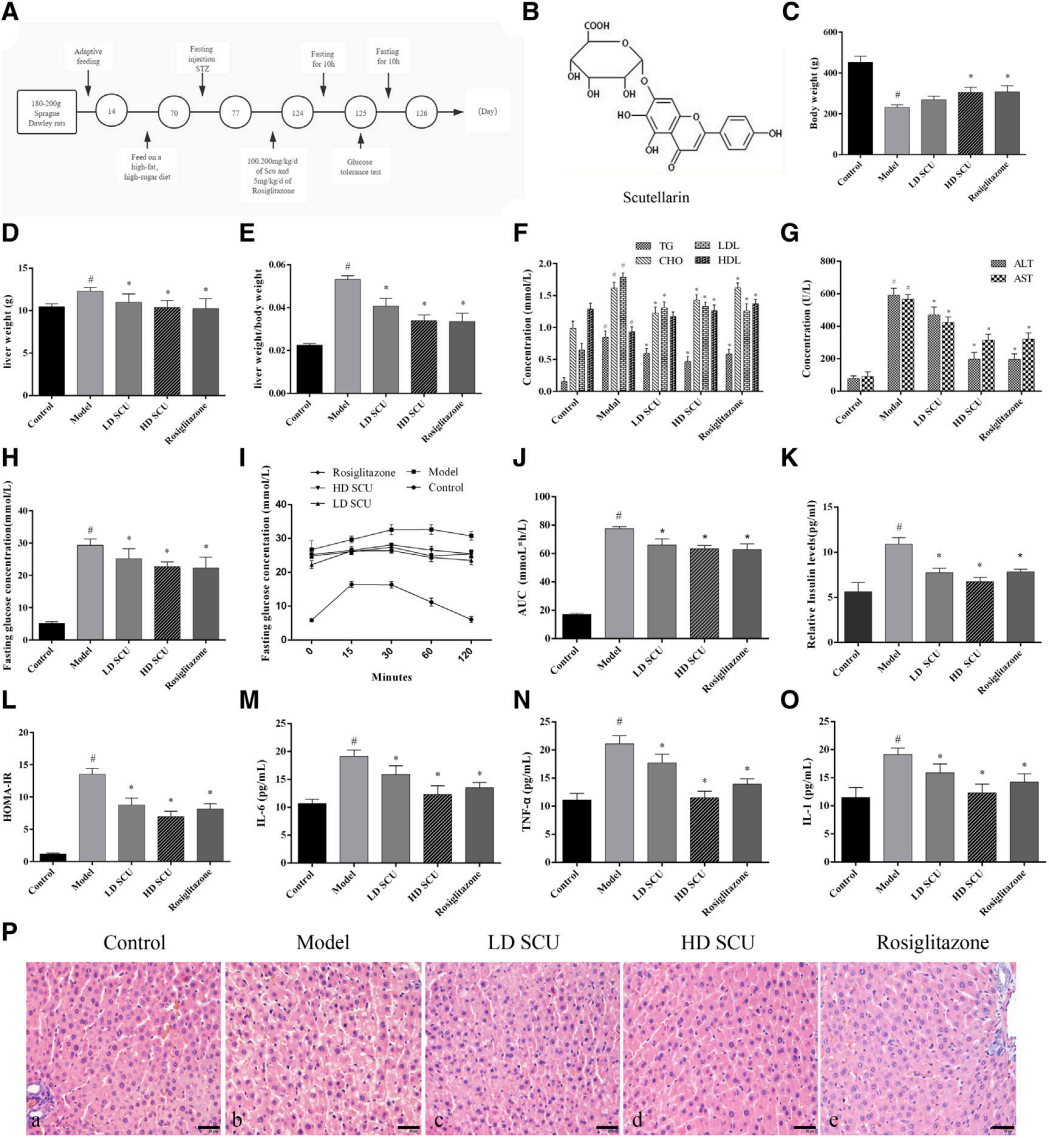
SCU can regulate blood glucose and insulin levels in type 2 diabetic rats. **(A)** Animal experiment process. **(B)** The molecular structure of SCU (Source from Guidechem, Hangzhou Dinghao Technology Co. LTD., China). **(C)** Body weight. **(D)** Liver weight. **(E)** liver/body weight. **(F)** The concentration of TG, CHO, LDL, HDL. **(G)** The concentration of ALT and AST. **(H)** The concentration of fasting blood glucose. **(I)** IPGTT of rats. **(J)** AUC of IPGTT. **(K)** Serum insulin levels. **(L)** Insulin resistance index. (**M**–**O)**. The concentration of IL-6. TNF-α, and IL-1. ^#^Represents the statistically significant difference from the normal group (*p* < 0.05), *Represents the statistically significant difference from the model group (*p* < 0.05). **(P)** HE staining of Liver tissue. **(A)** Normal group, **(B)** model group. **(C)** SCU low dose treatment group. **(D)** SCU high dose treatment group. **(E)** Rosiglitazone treatment group. *Represents the statistically significant difference from the model group (*p* < 0.05). Bar = 20 μm.

The Sprague Dawley rats were feed with high-fat and high-sugar (HFHS) diet, and injected with STZ to generate the type 2 diabetic models (Figure 1A). The structure of SCU was shown in Figure 1B. The diabetic rats were treated with SCU or Rosiglitazone which served as a positive control. We found that SCU can inhibit the increase of liver weight and liver weight/body weight induced by high-fat and high-sugar (HFHS) diet and STZ treatment (*p* < 0.05) (Figures 1C–E). Besides, SCU could improve the lipid metabolism by downregulating the level of serum triglyceride (TG), cholesterol (CHO), and low-density lipoprotein (LDL) and upregulating the level of high-density lipoprotein (HDL) induced by HFHS diet and STZ treatment (*p* < 0.05) (Figure 1F). Furthermore, SCU improve the liver function by inhibiting the level of alanine transaminase (ALT) and aspartate transaminase (AST) (Figure 1G). A similar finding was observed when treated with T2DM rats with Rosiglitazone (Figures 1C–F). Together, these results indicate that SCU is able to improve the dyslipidemia and liver function in type 2 diabetic rats.

### Scutellarin Regulates Blood Glucose and Insulin Levels in Type 2 Diabetic Rats

Next, the level of blood glucose was detected by Glucose meter. The results showed that the level of blood glucose was increased after HFHS diet and STZ treatment, while such increase was attenuated by SCU treatment. Rosiglitazone was also got the similar result with SCU (Figure 1H). Then the status of glucometabolic was detected by Intraperitoneal Glucose Tolerance Test (IPGTT) and the blood glucose area under the curve (AUC) was calculated by the formula: AUC = (FBG/2 + 1 h BG + 2 h BG/2) × 1 h mmolh/L. After an empty stomach injection of glucose, the blood glucose began to decrease in the normal group after 15 min. However, in the type 2 diabetes model group, the blood glucose concentration of the rats began to decrease after 60 min. After SCU treatment, the blood glucose began to decrease 30 min later. There was no significant difference between the SCU treatment group and the Rosiglitazone treatment group (Figures 1I,J).

Furthermore, the level of serum insulin and inflammatory factors were detected by ELISA kit. The results showed that SCU could inhibit the upregulation of the insulin induced by HFHS diet and STZ treatment (Figure 1K). At the same time, insulin resistance index (HOMA-IR) were calculated by the formula INS*GLU/22.5. Consistently, SCU treatment reduced the HOMA-IR value significantly (*p* < 0.05) (Figure 1L). SCU also inhibited the level of IL-6, TNFα and IL-1 induced by HFHS diet and STZ treatment. Interestingly, the high dose SCU group showed more effective than Rosiglitazone (Figures 1M–O). In addition, the histopathological changes of liver were evaluated by HE staining. In the normal group, the hepatic lobule structure was intact and the hepatocytes were arranged neatly. In the model group, the hepatocytes were arranged messily and the secretion of inflammatory factors was increased, while such disorder was improved by SCU treatment (Figure 1P). Together, these results showed that SCU could improve insulin resistance and inhibit inflammation of the liver in T2DM rats.

### Scutellarin Improves Oxidative Stress and Hepatic Apoptosis in Type 2 Diabetic Rats

**FIGURE 2:**
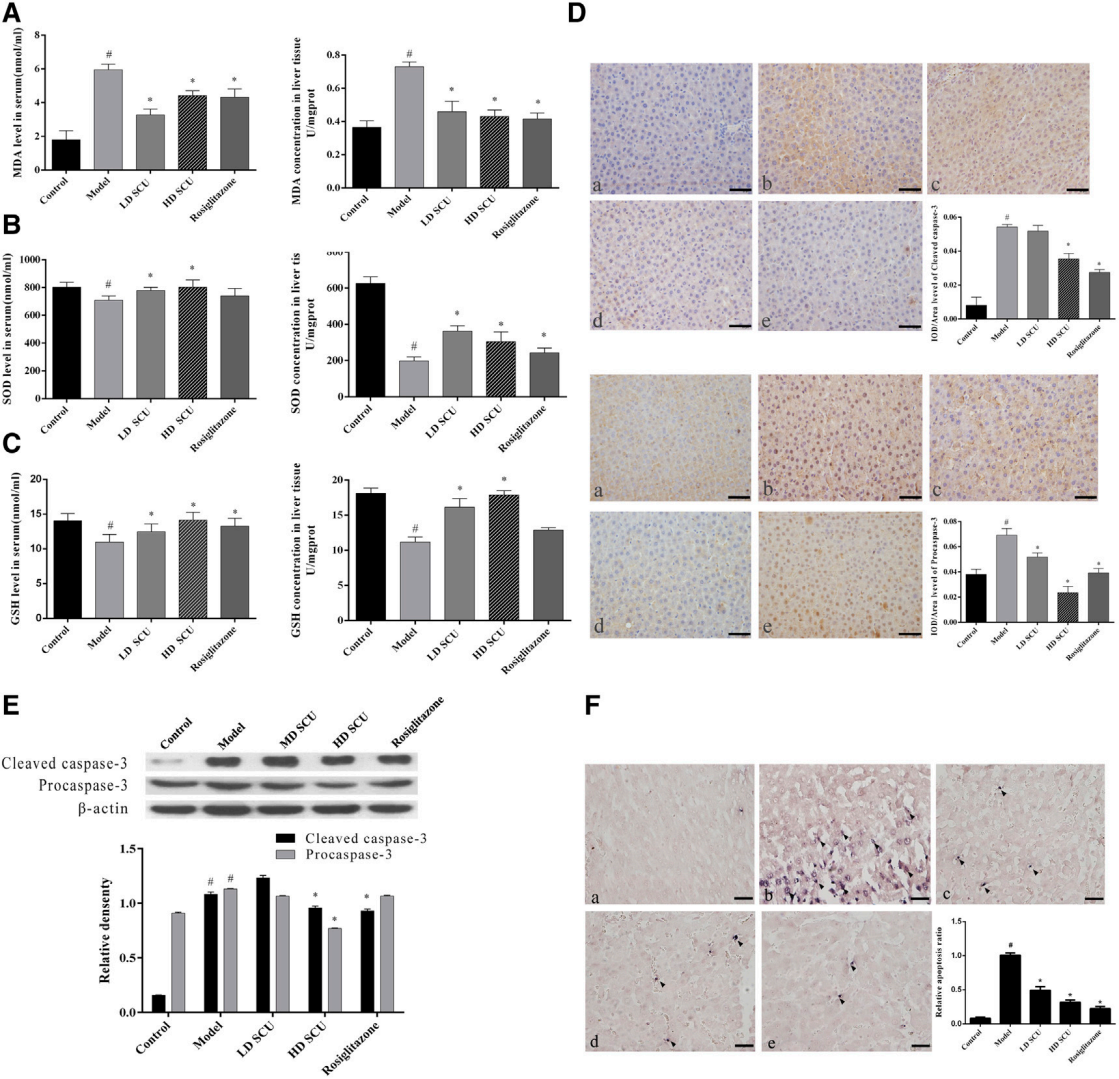
SCU can improve oxidative stress and hepatic apoptosis in type 2 diabetic rats. **(A)** MDA levels in serum and liver tissue. **(B)** SOD levels in serum and liver tissue. **(C)** GSH levels in liver tissue. **(D)** Immunohistochemical detection of Caspase-3. **(E)** Western blot detection of Caspase-3 (Procaspase-3 and Cleaved caspase-3). **(F)** TUNEL staining of liver tissue. **A**. normal group; **B**. model group; **C.** SCU low dose treatment group; **D.** SCU high dose treatment group; **E.** Rosiglitazone treatment group. ^#^Represents the statistically significant difference from the normal group (*p* < 0.05), *Represents the statistically significant difference from the model group (*p* < 0.05).

Then we investigate the effect of SCU treatment on regulation of the xidative stress and hepatic apoptosis in T2DM rats. The malondialdehyde (MDA) induces lipid peroxidation and is cytotoxic. It can reflect the degree of tissue peroxidation damage. The superoxide dismutase (SOD) is an antioxidant enzyme that can eliminate harmful substances produced in the process of metabolism. The glutathione (GSH) has antioxidant and detoxifying effects. The levels of MDA, SOD and GSH in serum and liver were measured. The results showed that the level of MDA was increased and the levels of SOD and GSH were decreased in the model group compared with the normal group (*p* < 0.05), suggesting an increased level of oxidative stress in T2DM rats. Interestingly, SCU and Rosiglitazone treatment normalized the levels of MDA, SOD and GSH in T2DM rats. It was worth to note that SCU showed better the therapeutic effect of on regulation of SOD and GSH than Rosiglitazone (Figures 2A–C). Since the oxidative stress can induce cell apoptosis, we then measured the level of Caspase-3 which is a key factor in the apoptotic pathway. The T2DM rats had a higher level of Caspase-3 protein than normal rats, while the SCU treatment reduced the Caspase-3 level in a dose dependent manner. It is worth to note that SCU conducted such effect better than Rosiglitazone did (Figures 2D,E). In addition, TUNEL staining showed that the T2DM rats had a significant number of apoptotic cells in their liver while no obvious apoptotic cells were observed in the liver of normal rats. Interestingly, SCU treatment resulted in a decreased number of apoptotic cells in T2DM rats in a dose dependent manner. Although Rosiglitazone treatment inhibited the cell apoptosis, it was less effective than high-dose of SCU (Figure 2F). Together, these results demonstrated that SCU improves oxidative stress and inhibits hepatic apoptosis in type 2 diabetic rats.

### Scutellarin Reduces the Level of Hcy in Type 2 Diabetic Rats

**FIGURE 3:**
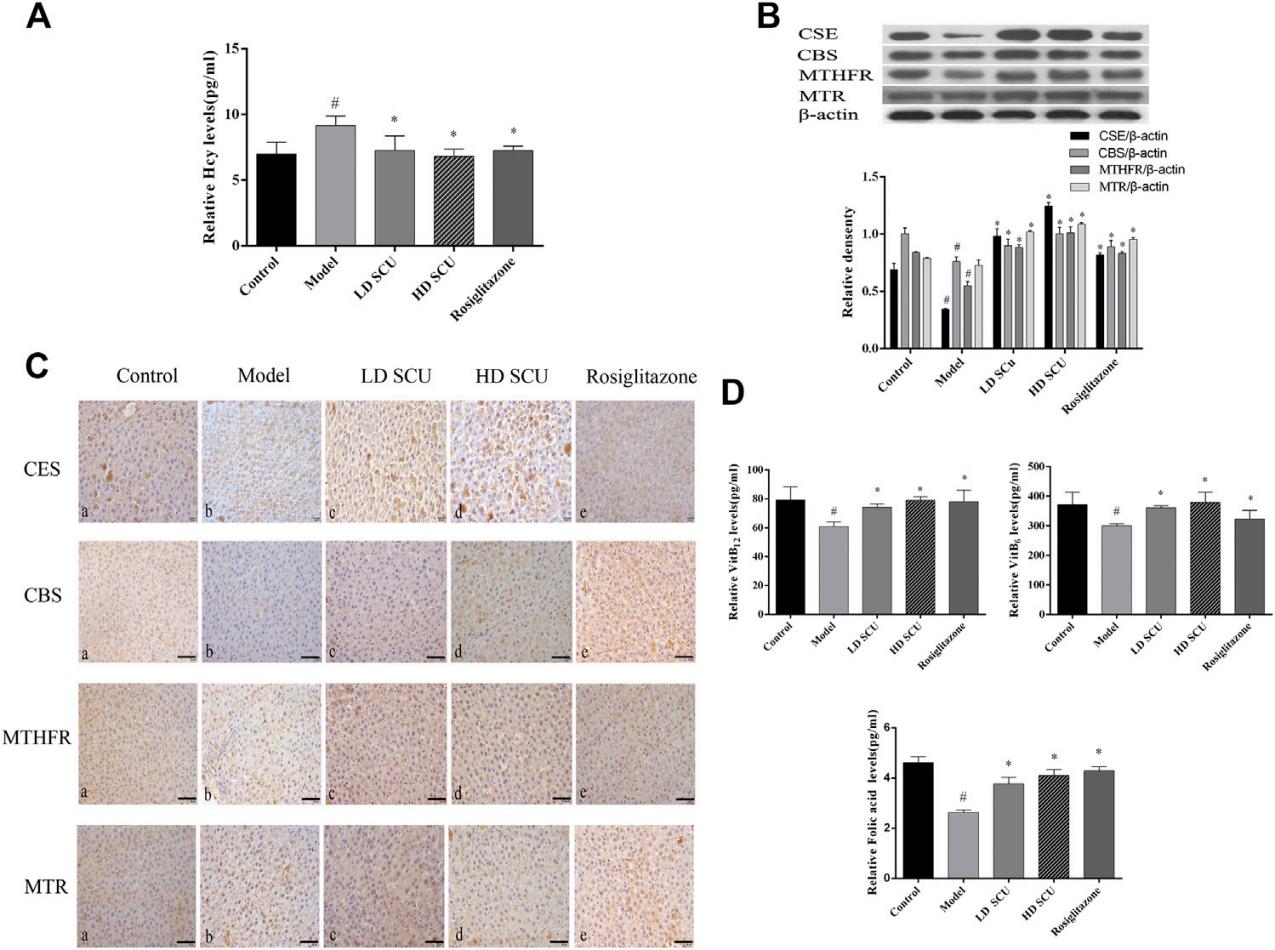
SCU can down-regulate Hcy levels in type 2 diabetic rats. **(A)** The levels of serum Hcy. **(B)** Western blot detection of CSE, CBS, MTHFR and MTR. **(C)** Immunohistochemistry results of CSE, CBS, MTHFR and MTR. **A**. normal group; **B.** model group; **C.** SCU low dose treatment group; **D.** SCU high dose treatment group; **E.** Rosiglitazone treatment group. **(D)** The level of Serum VitB6, VitB12 and folic acid. ^#^Represents the statistically significant difference from the normal group (*p* < 0.05), *Represents the statistically significant difference from the model group (*p* < 0.05).

High concentration of Hcy is closely associated with oxidative stress in type 2 diabetes mellitus. We found that the level of serum Hcy in the model group was increased compared with that in the normal group (*p* < 0.05), and it was decreased after SCU or Rosiglitazone treatment (*p* < 0.05). There was no difference in efficacy between SCU and Rosiglitazone (Figure 3A). The related enzyme and cofactors of Hcy metabolism were also detected by ELISA, immunohistochemical and western blot. CSE and CBS were related with the *trans*-sulfur pathway of Hcy metabolism. The results showed that the expression of CSE and CBS in the liver tissue of the model group was down-regulated compared with that in the normal group (*p* < 0.05). After SCU and Rosiglitazone treatment, the expression of CSE and CBS was up-regulated, and SCU has a stronger ability to regulate CSE than Rosiglitazone (*p* < 0.05) (Figures 3B,C). VitB_6_ as the cofactor of CSE and CBS was lower in the model group than in the normal group (*p* < 0.05), and increased after SCU treatment (*p* < 0.05), but Rosiglitazone did not have much control over the level of VitB_6_ (Figure 3D).

MTHFR and MTR were related with the methylation pathway of Hcy metabolism. The expression of MTHFR in the model group was lower than that in the normal group (*p* < 0.05), while the expression of MTR was comparable between the model group and the normal group. After SCU treatment, the expression of MTHFR and MTR was up-regulated (*p* < 0.05). The trend of immunohistochemistry and western blot was consistent (Figures 3B,C). The cofactors in the methylation pathway, folic acid and VitB_12_ were reduced in the model group (*p* < 0.05), and were elevated after treatment (*p* < 0.05), the therapeutic effects of SCU and Rosiglitazone were not much different (Figure 3D). Together, these results suggest that SCU might inhibit cell apoptosis through reducing the level of Hcy in T2DM rats.

### Scutellarin can Inhibit Apoptosis of LO2 Cells Induced by Hcy

**FIGURE 4:**
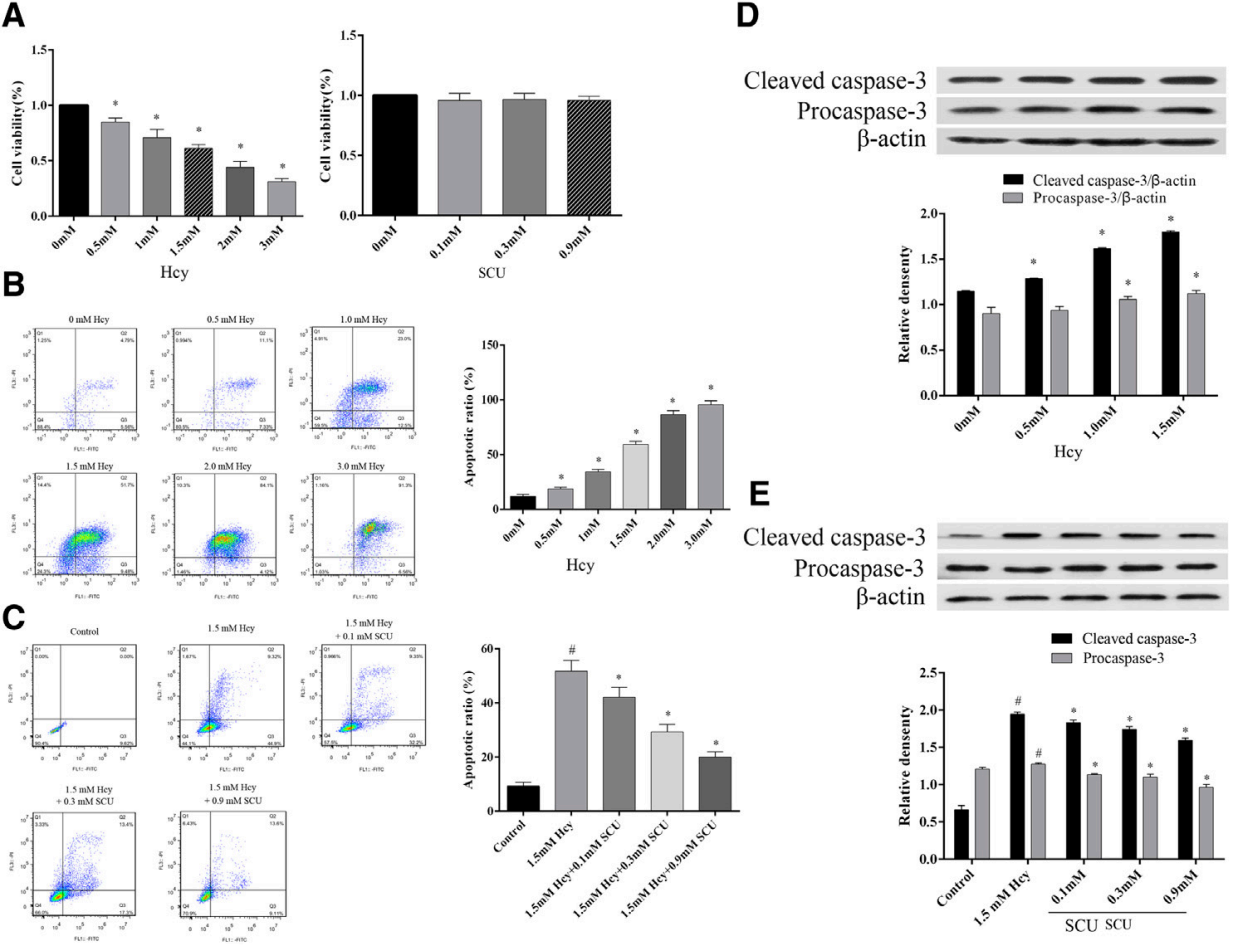
SCU can inhibit apoptosis of LO2 cells induced by Hcy. **(A)** The effect of Hcy and SCU on the LO2 cells viability. **(B)** The effect of Hcy on the LO2 cells apoptosis. **(C)** The effect of SCU on the Hcy-induced LO2 cells apoptosis model. **(D)** The effect of Hcy on Caspase-3 in LO2 cells. **(E)** The effect of SCU on Hcy-induced LO2 cells apoptosis model. ^#^Represents the statistically significant difference from the normal group (*p* < 0.05), *Represents the statistically significant difference from the model group (*p* < 0.05).

To determine the influence of SCU on apoptosis induced by Hcy, the LO2 cells were treated with Hcy and SCU. The results showed that Hcy inhibit the proliferation activity of LO2 cells in a dose-dependent manner, in contrast, while SCU has no significant influence on the cytotoxicity of LO2 cells by CCK8 assay (Figure 4A). Meanwhile, the apoptosis of LO2 cells were significantly induced by Hcy in a dose dependent manner (Figure 4B), and SCU can reverse that change in a dose dependent manner by flow cytometry analysis (Figure 4C). In addition, Hcy upregulated the expression of Cleaved caspase-3, while this upregulation was repressed by SCU treatment in the LO2 cells (Figures 4D,E). The above results showed that SCU can inhibit apoptosis of LO2 cells induced by Hcy.

## Discussion

Hcy is one of the independent risk factors for the development and progression of atherosclerosis in the body. As a sulfur-containing amino acid, the mechanism of Hcy metabolism in the body mainly through the remethylation pathway and the sulfur transfer pathway. In the remethylation pathway, methionine synthase (MTR) catalysis is required, with VitB_12_ as a cofactor, which can make 5-methyl-tetrahydrofolate with folic acid as a cofactor. Meanwhile, the MTHFR catalyzes the conversion to tetrahydrofolate, and the methyl is bind by Hcy to form methionine. In the sulfur transfer pathway, CBS with VitB_6_ as a cofactor converts homocysteine to cystathionine. Cysteine and α-ketobutyrate are further produced by the action of CSE (Taysi et al., 2015). The changes of Hcy levels are affected by genetic and non-genetic factors, and MHTFR gene defects can lead to elevated levels of Hcy (Tang et al., 2017). Abnormalities in folic acid, VitB_6_ and VitB_12_ in the diet can also lead to an increase of Hcy levels (Caldeira-Araujo et al., 2019). A large number of studies have shown that Hcy is increasing in type 2 diabetes. Some scholars have found that Hcy can also cause mitochondrial dysfunction in human umbilical vein endothelial cells, activate the PERK pathway, and cause apoptosis (Zhang et al., 2017c). At the same time, Hcy also induces endoplasmic reticulum stress, thereby strengthening the umbilical vein endothelial cells in patients with type-2 diabetes mellitus (Mao et al., 2016).

Therefore, it is necessary to find an agent of lower toxicity to target the metabolism of Hcy and alleviate liver injury in type 2 diabetes. Natural compound has low toxicity and high efficacy. It is very interesting to search for the pharmaceutical values of a natural compound in chemoprevention and chemotherapy (Feng et al., 2012). SCU is considered to be less toxicity (Chan et al., 2012; Uzuner et al., 2012). In addition, we also found that SCU at 1mM had no effect on cell activity (Figure 4A). At present, tens of millions of patients use SCU and related pharmaceutical preparations every year in China. Previous studies have shown that SCU can treat and prevent diabetes and its complications (Jiang et al., 2016; Chledzik et al., 2018). It was reported that SCU can inhibit hyperglycemia-induced apoptotic cells and morphologic impairments in testes of diabetic rats (Long et al., 2015). SCU could regulate myocardial Ca (^2+^)-cycling proteins and have protective effect on diabetic cardiomyopathy (Wang et al., 2010). Besides, SCU could inhibit high glucose-mediated vascular inflammation (Luo et al., 2008). In contrast, HHcy cause the oxidized stress and inflammation and promote vasculopathy in the complication from type 2 diabetes. (Doupis et al., 2010; Hu et al., 2019; Tawfik et al., 2019). However, the effects of SCU on hepatocyte apoptosis have not been studied above in type 2 diabetes. By studying the effect of SCU on Hcy metabolism, we studied the effect of SCU on hepatocyte apoptosis in type 2 diabetes *in vitro* and *in vivo*.

In our studies, we found that SCU can decrease the levels of serum TG, CHO, glucose, insulin and improve insulin resistance induced by HFHS diet and STZ treatment. SCU can inhibit oxidative stress and apoptosis of liver tissue and cells, and then attenuate HFHS and STZ-induced pathologic change of liver. The specific molecular mechanisms of by which SCU improve type 2 diabetes model were to upregulate the key enzyme of Hcy metabolism MTHFR, MTR, CBS and CSE with their cofactor VitB_12_, VitB_6_ and folic acid. The apoptosis mechanism of type 2 diabetic hepatocytes is relatively complex, and more studies on SCU in type 2 diabetic liver disease are needed in the future. In conclusion, SCU promoted the clearance of Hcy and improved HFHS and STZ-induced hepatic impairment.

## Data Availability Statement

The datasets generated for this study are available on request to the corresponding authors.

## Ethics Statement

The Animal study was reviewed and approved by Experimental Animal Center of Kunming Medical University.

## Author Contributions

YW and XF performed research, analyzed data, and wrote the paper; BF, HZ, DG, and HS performed animal research; KJ and FK performed cell experiment and analyzed data; ShuL and ShaL designed research and analyzed data.

## Funding

This work was supported by grants from National Natural Science Foundation of China (81360128). Scientific and Technological Development Project of Yunnan Province (2018FE001-162,2017FE467). Education Department Fund of Yunnan Province (2017YJS076). Health research project of Kunming Municipal Health Commission (2019-03-10-008). Kunming Health science and technology personnel training project (“ten hundred thousand” project). This work was also supported by Yunnan Province Key Laboratory for Nutrition and Food Safety in Universities.

## Conflict of Interest

The authors declare that the research was conducted in the absence of any commercial or financial relationships that could be construed as a potential conflict of interest.
